# Development of a Threshold Model to Predict Germination of *Populus tomentosa* Seeds after Harvest and Storage under Ambient Condition

**DOI:** 10.1371/journal.pone.0062868

**Published:** 2013-04-26

**Authors:** Wei-Qing Wang, Hong-Yan Cheng, Song-Quan Song

**Affiliations:** Key Laboratory of Plant Resource, Institute of Botany, The Chinese Academy of Sciences, Beijing, China; Lawrence Berkeley National Laboratory, United States of America

## Abstract

Effects of temperature, storage time and their combination on germination of aspen (*Populus tomentosa*) seeds were investigated. Aspen seeds were germinated at 5 to 30°C at 5°C intervals after storage for a period of time under 28°C and 75% relative humidity. The effect of temperature on aspen seed germination could not be effectively described by the thermal time (TT) model, which underestimated the germination rate at 5°C and poorly predicted the time courses of germination at 10, 20, 25 and 30°C. A modified TT model (MTT) which assumed a two-phased linear relationship between germination rate and temperature was more accurate in predicting the germination rate and percentage and had a higher likelihood of being correct than the TT model. The maximum lifetime threshold (MLT) model accurately described the effect of storage time on seed germination across all the germination temperatures. An aging thermal time (ATT) model combining both the TT and MLT models was developed to describe the effect of both temperature and storage time on seed germination. When the ATT model was applied to germination data across all the temperatures and storage times, it produced a relatively poor fit. Adjusting the ATT model to separately fit germination data at low and high temperatures in the suboptimal range increased the models accuracy for predicting seed germination. Both the MLT and ATT models indicate that germination of aspen seeds have distinct physiological responses to temperature within a suboptimal range.

## Introduction

*Populus tomentosa* is a native *Populus* species which distributed mainly in the middle and lower reaches of Yellow River in the north of China [1]. The *Populus* genus is a common species dominating the riparian woodland ecosystem [2]–[5]. Though a single mature female *Populus* can produce thousands or even millions of cottony seeds in most years [6], the recruitment of new individuals is rare under the natural environment [7]–[10]. Many studies have indicated that the successful regeneration of aspen seeds is extremely dependent on bare and moist soil conditions created by flood events in the riparian woodland ecosystem. Human activity, such as river regulation and water reclamation projects, further destroy this ecosystem and decrease the probability of seed germination [4], [8]–[10]. Under the natural environment, an optimal condition for seed germination is only sustained for a short period of time due to the limitation of temperature, moisture, and other environmental factors. Therefore, the time to germination might be one of the key factors determining the successful regeneration of aspen seeds.

Temperature is the most important environmental factor in regulating the percentage and rate of seed germination. Effect of temperature on seed germination has been successfully predicted and characterized by the thermal time (TT) model [11]–[16]. Generally, this model assumes that at a suboptimal range of temperatures, the base temperature for germination (*T_b_*) is constant and the thermal time required for germination of a given fraction of seeds (*θ_T_*(*g*)) is a normal or log normal distribution among seeds in a population; while at a supraoptimal range, the maximum temperature for the germination of a given percentage g (*T_c_*(g)) distributes normally, and the thermal time is constant among seeds in a population [16]–[21]. However, this general assumption does not apply to all species, for example, rangeland grass species [14], [22], [23], tropical pasture species [24] and *Lithospermum arvense* [15]. In these species, other assumptions, such as piece-wise linear relationships between germination rates and temperature [24], [25] and normal distribution of *T_b_* and constant *θ_T_*(*g*) [15] have been suggested to predict accurately germination rates and/or percentages.

In the *Populus* species, it has been found that the seed longevity is very short and is limited to several days or weeks under an ambient environment [5], [8], [26], [27]. Thus, seed viability is also an important factor determining the timing of aspen seed germination. Relationships between the time and percentage of germination and loss of seed viability can be well described by the maximum lifetime threshold (MLT) model [28]. This model assumes that there is a maximum potential lifetime for each seed, which distributes normally among individual seed in a population, and that the time to germination of a given seed is inversely proportional to the difference between the aging time and the maximum potential lifetime of that seed [28]. This approach has accurately predicted the germination times and percentages of lettuce (*Lactuca sativa* L.) seeds aged for a series of periods of time [28]. However, this model has been rarely evaluated in other species.

In the present study, we investigated germination behavior of *P. tomentosa* seeds at various temperatures before or after storage for a series of periods of time under ambient conditions. It aimed to test validity of the TT and MLT model in describing the effect of temperature and storage time on aspen seed germination, respectively. Based upon the TT and MLT model, we further developed a model that could describe the effect of combined factors of both temperature and storage time on seed germination. This model would be useful to predict germination time and percentage of aspen seeds across all suboptimal temperatures and storage times at which germination of aspen seeds can occur. These models may help explain some of the reasons why aspen seeds cannot regenerate successfully under natural conditions.

## Materials and Methods

### Ethics Statement

No specific permits were required for the described field studies. The location is not privately-owned or protected in any way, and the field studies did not involve endangered or protected species.

### Seed Collection

The period of aspen (*P. tomentosa*) seed dispersal was from April 25 to May 20, and the peak time was between May 1 and 15 in 2009 in the Beijing Botanical Gardens (N 39°59′, E 116°13′; altitude, 73 m), Xiangshan, Beijing, China. The mature aspen seeds with cotton were collected on May 12, 2009.

### Water Content Determination

Four replicates of 100 seeds each were sampled for water content determination according to the International Seed Association Rules [29]. Water content of seeds was expressed on a basis of fresh weight.

### Seed Storage

Seeds with cotton were dried at 28±2°C and 75±5% relative humidity (RH) for 2 days, the water content of seeds reaches to about 9.9%. These seeds were stored under the same condition mentioned above for 0 (control seed), 5, 10, 15, 20, 25, 45 and 65 days. After storage, the seeds were excised from the cotton by hand and used for germination.

### Seed Germination

After storage for different periods of time, the seeds were sampled and germinated. Three replicates of 50 seeds each were germinated on two layers of filter paper moistened with 4 ml of distilled water in closed 7-cm-diameter Petri dishes in darkness at 5, 10, 15, 20, 25 and 30°C. Distilled water was added to the filter paper each day to maintain constant moisture during germination. Germination was recorded per hour or day. Radical protrusion to 1 mm was used for criterion of germination. Germination was stopped when no germination of seeds was observed within 7 days. Times required to achieve 10, 30, 50 and 70% germination were calculated for control seeds by linear interpolation between daily germination percentiles from the cumulative germination curves [17].

### Viability Test

After storage for the given periods of time, three replicates of 25 seeds each were stained in 0.5% 2,3,5-triphenyl tetrazolium chloride (TTC) solution at 25°C to test seed viability. The viability of non-germinated seeds incubated at different temperatures was also tested by the TTC staining.

### Model Definition and Statistical Analysis

#### Thermal time (TT) model

This model assumes that germination rate (1/*t_g_*) for a given germination percentile (g) is a linear function of temperature (*T*) at a suboptimal range,
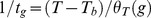
(1)
*θ_T_*(*g*) is the thermal time to germination of a given percentage g and *T_b_* is the base temperature.

In many cases, *T_b_* is assumed to be constant, and *θ_T_*(*g*) to be normally or log normally distributed among seeds in the population. It was found that assumption of a log normal distribution of *θ_T_*(*g*) was applicable for germination prediction of aspen seeds, i.e.,
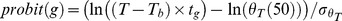
(2)(*θ_T_*(*50*) is the mean of *θ_T_*(*g*), and *σ_θT_* is the standard deviation of *lnθ_T_*(*g*)).

#### Maximum lifetime threshold (MLT) model

This model assumes that there is a linear relationship between germination rate (1/*t_g_*) and aging time (*p*):
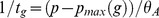
(3)where *p_max_*(*g*) is the maximum potential lifetime above which seed germination cannot occur for a given percentage g and is assumed to be normally distributed among seeds in a population; and *θ_A_* is an “aging time constant”. Based upon this assumption, germination response to storage treatment is characterized by the following probit equation:
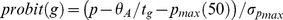
(4)(*p_max_*(*50*) and *σ_pmax_* are the mean and standard deviation of *p_max_*(*g*)).

According to equation (3), it can be used a factor of (1-*p*/*p_max_*(*g*))*t_g_*(*p*) to normalize the germination time of a seed fraction at any storage time to the corresponding germination time that would occur in the control seeds [28], i.e.,
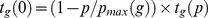
(5)


This equation removes the effect of increasing storage time on the germination time course. If application of this factor normalizes the time courses at different storage times to a common predicted time course, it indicates that the MLT model parameters have accurately described the sensitivity of germination to storage time in a seed population [28].

#### Aging thermal time model

In this model, a parameter of aging thermal time (*θ_AT_*) that combined the thermal (equation (1)) and aging time (equation (5)) at suboptimal range of temperature is defined:
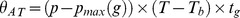
(6)


When *θ_AT_* is assumed to be constant, and *p_max_*(*g*) and *T_b_* to be constant and independent of temperature and aging time, respectively, seed germination at a given temperature and storage time can be predicted according to:
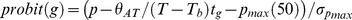
(7)


For the assumption of constant *θ_AT_* and *T_b_*, it is facilitated to be evaluated by the following equation:
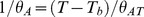
(8)


This equation was rewritten according to equation (3) and (6). Thus, if plotting of 1/*θ_A_* versus germination temperature follows a linear relationship, the *θ_AT_* and *T_b_* would be expected to be constant.

Using the germination time normalization factor (equation (5)), a normalized thermal time (*θ_NT_*) is calculated:
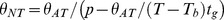
(9)


At this time, the equation (7) can be rewritten to:
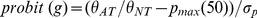
(10)


This equation indicates that if seed germination can be related to the normalized thermal time by a common fitting curve, the ATT model describes accurately the effect of both temperature and storage time on seed germination.

### Statistic Analysis

The ‘nls’ function of R program [30] was chosen for the repeated linear regression with probit germination versus germination temperature and time (equation (2) and (13)), aging and germination times (equation (4)) or aging time, germination time and temperature (equation (7) and (15)). The function estimates parameters through an iterative least square procedure. Graphpad Prism 5.0 (GraphPad Software) was applied for graph plotting and data regression with constraint parameters. It should be noted that the final 5% of germination of each treatment was excluded from the regression.

Models were compared for their likelihood using the Akaike Information Criterion (AIC), that is, the lower the AIC value, the greater the likelihood of the model [31]. The adjusted *R* square (*R^2^*) and root mean squared error (*RMSE*), a common criterion to quantify the mean difference between simulation and measurement [32] were adopted to be criterion for goodness of fit. The *RMSE* is defined that:
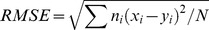
(11)where *n_i_*, *x_i_*, and *y_i_* are the number of observed data, the simulation data, and the mean observed data of treatment i, respectively, and N is the total number of observed data.

## Results

### Effect of Temperature on Seed Germination

**Figure 1:**
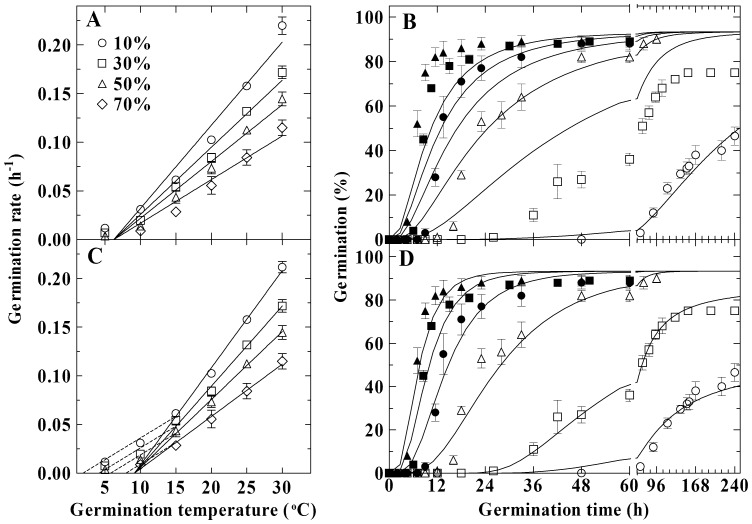
Comparison between the thermal time (TT) and the modified thermal time (MTT) models in describing germination of control seeds at various temperatures. TT model: germination rates were regressed with linear lines constrained to converge upon a common base temperature (A), and time courses of germination at 5 (○), 10 (□), 15 (△), 20 (•), 25 (▪) and 30 (▴)°C were fit to equation (2) according to parameters in Table 1 (B). MTT models: germination rates were regressed with lines constrained so they shared the same slope at temperature range of 5–10°C and to converge upon a common base temperature at 15–30°C (C), and the time course of germination at 5–10°C and 15–30°C were fit to equations (13) and (2) according to parameters in Table 1, respectively (D).

In control seeds, representative germination rates (the inverse of times to germination of a given percentage g, 1/*t_g_*) for 10, 30, 50 and 70% of germination were plotted against germination temperature to show the trend of change in germination rates with temperature (Figures 1A and C). These rates showed typically continuous increase with temperature (Figures 1A and C). Based upon the TT model, this trend of change was fitted with linear lines constrained to be converged to a common *T_b_* (Figure 1A). These lines appear to fit well the germination rates at 10–30°C, but not the rates at 5°C (Figure 1A). Furthermore, when the TT model (equation (2)) was fitted to the complete germination data across all the temperatures, it poorly predicted the time course of germination at 10, 20, 25 and 30°C, at which the actual data (symbols) deviated from the predicted germination percentage (curves) (Figure 1B).

A modified TT (MTT) model was developed to more accurately predict germination rates and percentage of aspen seeds. It was assumed that the increase in germination rates with temperature followed a two-phased linear relationship: (1) at 15–30°C, the linear relationship also conformed to the assumption of the TT model, i.e., germination rates for all percentiles versus temperature were linearly converged to a common *T_b_* value (Figure 1C); and (2) at 5–10°C, the linear lines for the relationship between germination rate and temperature were extrapolated to different ‘base’ temperatures (to differentiate from the *T_b_* in equation (2), this temperature was defined as the minimum temperature for germination, *T_m_*) with a common slope of 1/*θ_Tm_* (Figure 1C), i.e.,
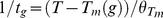
(12)where *T_m_*(*g*) is the minimum temperature below which seeds cannot germinate to a given percentage g and is assumed to be normally distributed among seeds in a population, and *θ_Tm_* is defined to be the thermal time for seed germination and is assumed to be constant.

At this time, complete germination data at 15–30°C were fit to the TT model (equation (2)), while at 5–10°C, those were modeled according to the normal variation in *T_m_*(*g*) values:
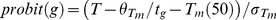
(13)(*T_m_*(*50*) and *σ_Tm_* are the mean and standard deviation of *T_m_*(*g*), respectively).

The MTT model increased the *R^2^* and decreased *RMSE* value (Table 1), and improved the model accuracy in predicting germination rates (Figure 1C) and time courses of germination in comparison to TT model (Figure 1D). In addition, the AIC value suggested that the MTT model had a higher likelihood to be correct when compared to the TT model in describing the effect of temperature on seed germination, especially those at 5–10°C (Table 1). Furthermore, the MTT model could more accurately predict germination of stored seeds than the TT model (Supplemental Table S1).

**Table 1:** Comparison between the thermal time (TT) and the modified thermal time (MTT) model in describing germination of control seeds (0 day of storage) at various suboptimal temperatures.

	5–10°C		15–30°C	
	TT	MTT	TT	MTT
*θ_Tm_* or *θ_T_*(*_50_*) (°C h)	253.7	383.9	253.7	149.1
*T_m_*(*50*) or *T_b_* (°C)	3.9	4.4	3.9	9.5
*σ_Tm_* (°C) or *σ_θT_* (°C h)	0.79	3.79	0.79	0.56
*R^2^*	0.77	0.99	0.88	0.94
*RMSE*	0.129	0.029	0.139	0.098
AIC	–246	–358	–271	–292

h, hours.

### Effect of Storage Time on Seed Germination

**Figure 2:**
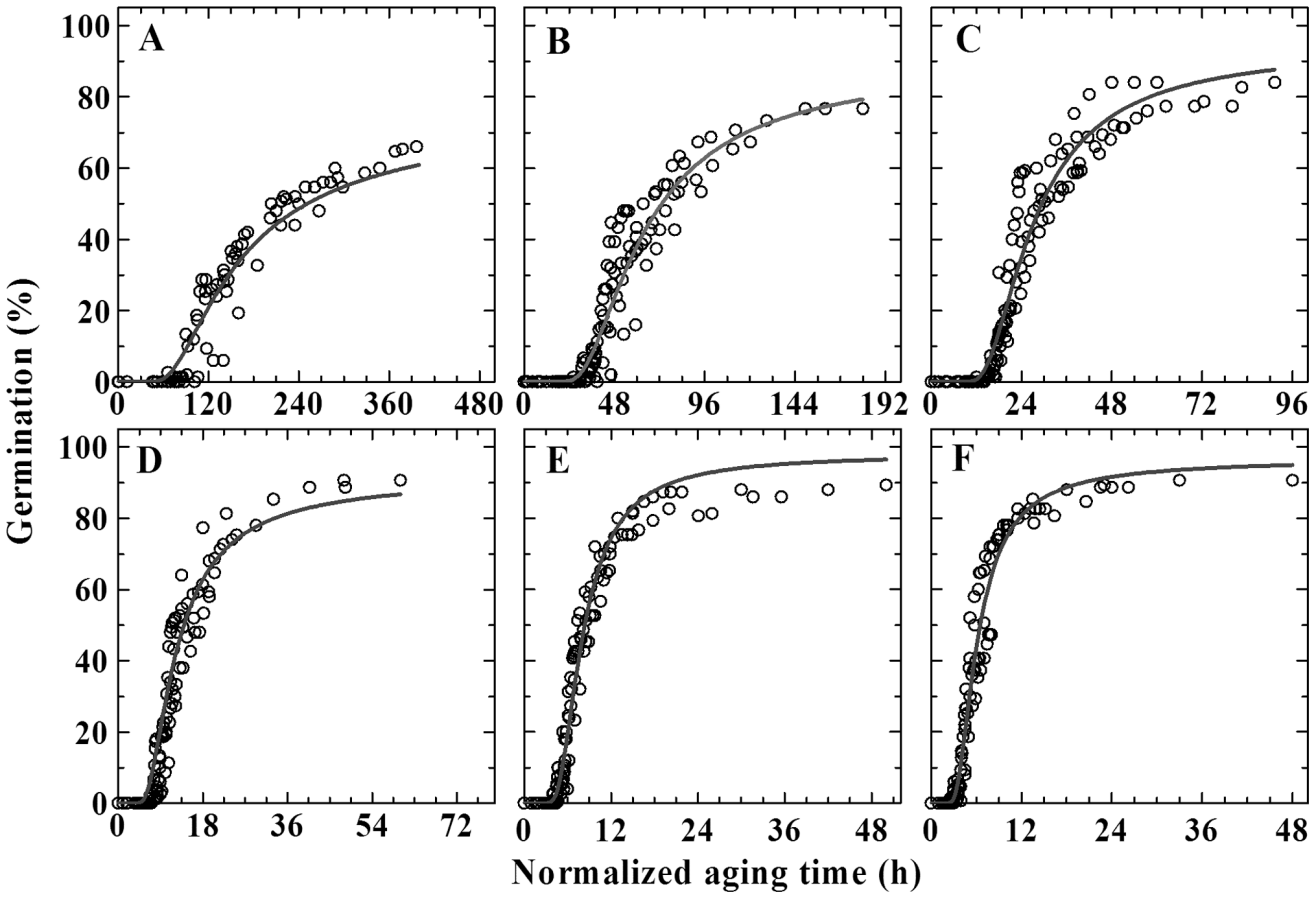
Normalized time course of germination for seeds at various temperatures. The actual times of germination for seeds stored for 5, 10, 15, 20, 25, 45, and 65 days at a given temperature of 5 (A), 10 (B), 15 (C), 20 (D), 25 (E) or 30°C (F) were normalized to the time course for control seeds at that temperature according to equation (5) and then plotted against germination percentage of seeds. The empty circles are the actual data and the gray curves are predicted from equation (4) with parameters in Table 2.

The effect of storage time on seed germination was characterized by the MLT model (equation (4)). Except for a relatively poor fit at 5°C (*R^2^* = 0.66), the MLT model accurately described the effect of storage time on seed germination (*R^2^* from 0.81–0.91, Table 2). When time courses of germination at a given temperature after storage were normalized to the time course of control seeds using a factor of (1-*p*/*p_max_*(*g*))*t_g_*(*p*) (equation (5)), germination data at each germination temperature were well predicted by a common curve (Figure 2).

**Table 2:** Parameters of the maximum lifetime threshold (MLT) model described the germination of aspen seeds across a series of storage periods of time at a given temperature (equation (4)).

	Germination temperature (°C)					
	5	10	15	20	25	30
*p_max_(50)* (d)	17.45	26.55	32.88	30.54	31.32	27.86
*σ_pmax_* (d)	23.54	19.30	19.30	22.43	14.44	14.72
*θ_A_* (d h)	4384.03	1940.62	966.18	454.34	262.05	181.06
*R^2^*	0.66	0.82	0.91	0.81	0.91	0.87

d, days; h, hours.

### Effect of both Temperature and Storage Time on Seed Germination

**Figure 3:**
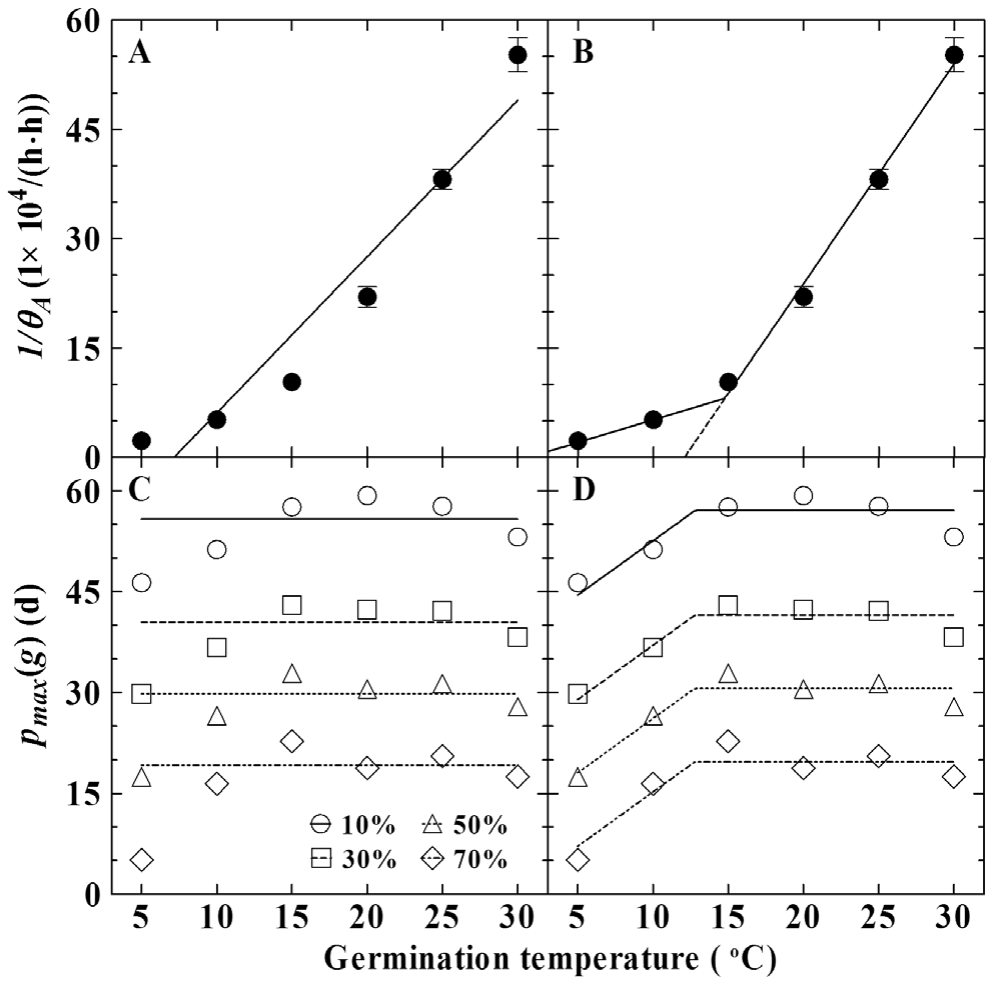
Correlation of *1/θ_A_* and *p_max_*(*g*) with germination temperature. *1/θ_A_* and reprehensive *p_max_*(*g*)s for 10, 30, 50 and 70% estimated from the MLT model were plotted against temperature to show trend of change in these two parameters. *1/θ_A_* was related to all the germination temperatures with a linear line (A, R^2^ = 0.926) or related separately to temperatures at 5–10°C and 15–30°C with two different linear lines (B, *R^2^* = 0.996). *p_max_*(*g*) was assumed to be constant across all the germination temperatures (A) or to be constant at 15–30°C and declined linearly with temperature decreased at 5–10°C (B, *R^2^* = 0.914).

**Figure 4:**
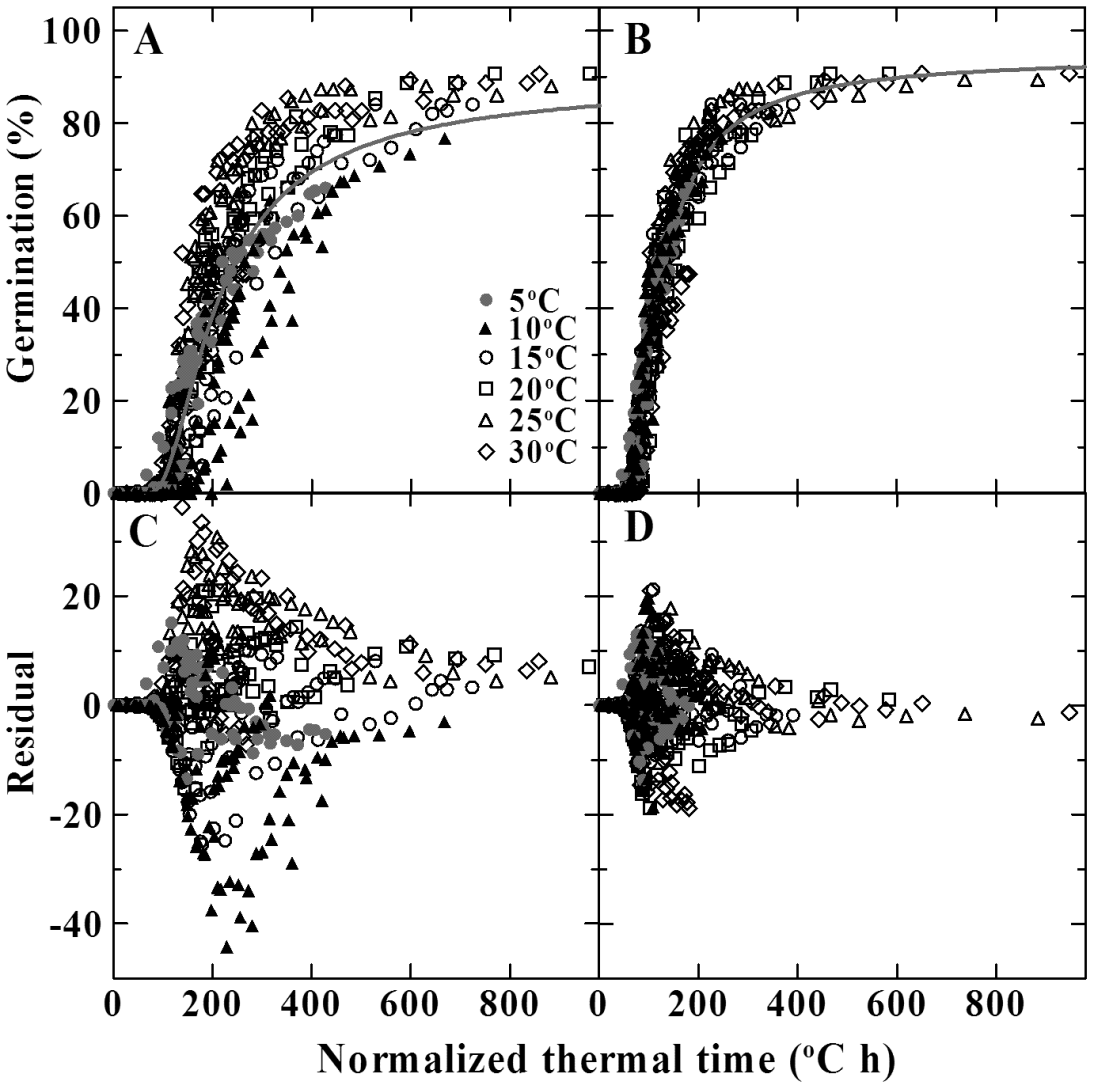
Normalized thermal time courses of seed germination at different temperatures and storage times. The time courses of germination at different temperatures and storage times were plotted on a normalized thermal time scale (A and B). These normalized thermal times were calculated according to equation (9) (A) and equation (9) and (14) (B) using the one-phased and two-phased model parameters in Table 3, respectively. Regression lines (gray lines) were plotted using a common set of constants in one-phased model (A), and using the parameters at 15–30°C in two-phased model (B) shown in Table 3. The residuals of the regressions were shown in C and D.

Because the TT model could not accurately predict germination across all the suboptimal temperatures, it would be expected that the ATT model (equation (7)) on the basis of the TT model could also not accurately predict germination in this range. When the estimated 1/*θ_A_* (Table 2) was plotted and correlated to the germination temperature, a linear relationship was obtained (*R^2^* = 0.926, Figure 3A). However, this regression did not fit the data at 5°C (Figure 3A), where the *p_max_*(*g*)s deviated largely from the values at other temperatures (Figure 3C). At the same time, when equation (7) was used to fit germination data across all the germination temperatures and storage times, the data fit poorly, as can be seen from large deviation of predicted germination from the actual data (Figures 4A and C). Thus, as the TT model, the ATT model was modified to separately fit the germination data at 5–10°C and 15–30°C.

At 15–30°C, a linear regression on 1/*θ_A_* with temperature closely matched the trend of change in 1/*θ_A_* (Figure 3B). Plotting of representative *p_max_*(*g*)s for 10, 30, 50 and 70% germination versus temperatures showed that the *p_max_*(*g*)s kept relatively constant in this range (Figure 3D). These results agreed well with the assumption on constant parameters of 1/*θ_AT_*, *T_b_* and *p_max_*(*g*) for the ATT model. When germination data at 15–30°C across all the storage times were fit with the equation (7), it worked well (R^2^ = 0.87) for describing the effect of both temperature and storage time on seed germination in this temperature range (Table 3, Figures 4B and D). Using the model parameters in Table 3 and equation (9), the time courses of germination at 15–30°C and all the storage times were plotted on a common normalized thermal time scale and all fit a common predicted curve (Figures 4B and D).

**Table 3:** Parameters of the aging thermal time (ATT) model describing the germination of aspen seeds across a range of temperatures and storage periods of time.

	One-phased model		two-phased model	
	5–10°C	15–30°C	5–10°C	15–30°C
*p_max_*(*50*) (d)	25.53	25.53	30.79	30.79
*θ_AT_* (d°C h)	6261	6261	18114	4158
*σ_pmax_* (d)	21.58	21.58	22.5	18.05
*T_b_* (°C)	3.99	3.99	1.49	10.26
*k* (d°C^−1^)	–	–	1.41	–
*T_i_* (°C)	–	–	12.06	–
*R^2^*	0.84	0.72	0.96	0.87
*RMSE*	0.1647	0.1389	0.0868	0.0949
AIC	–1025	–2781	–1391	–3320

One phased model: the ATT model was fit against germination data across all the suboptimal temperatures and storage times; two-phased model: the ATT model were fit against germination data at 5–10°C and 15–30°C and all the storage times, respectively. d, days; h, hours.

At 5–10°C, it was assumed that 1/*θ_A_* versus temperature also followed a linear relationship, but had a different slope and intercept, i.e., *θ_AT_* and *T_b_* values from those at 10–30°C (Figure 3A) and *p_max_*(*g*) were a linear function of *T* below a given temperature *T_i_* (Figure 3B), i.e.,
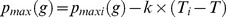
(14)(*p_maxi_*(*g*) is the *p_max_*(*g*) value at *T_i_*, *k* is the rate of decrease in *p_max_*(*g*)). Based upon these linear relationships, the ATT model (equation (7)) was modified to:
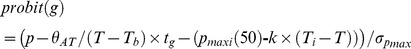
(15)where *p_maxi_*(*50*) is the *p_max_*(*50*) value at *T_i_*, and is initially estimated through equation (7). This equation described well (R^2^ = 0.96) the germination response to storage time and temperature at 5–10°C (Table 3, Figures 4B and D). In equation (14), the *p_maxi_*(*g*) is equal to *p_max_*(*g*)+*k*×(*T_i_*-*T*). Using this factor, the time courses of germination at 5–10°C and all the storage times could be normalized to the equivalent time course of control seeds at *T_i_*, and after multiplied by a factor of (*T_i_*-*T_b_*), these data were plotted on a common normalized thermal time scale (Figure 4B). The normalized thermal time courses coincided with the normalized data at 15–30°C and were closely matched by a same predicted curve for those at 15–30°C (Figures 4B and D).

As the TT model, the two-phased ATT model is more accurate in predicting seed germination and had a higher likelihood to be correct than the one-phased model (Table 3, Figure 4).

## Discussion

In many cases, the assumption of the TT model in the suboptimal range of temperatures is that the *T_b_* is constant and the thermal time to germination of a given percentage g (*θ_T_*(g)) is normal or a log normal distribution within a given seed population [11], [13], [16]–[18], [20], [21]. However, when we accepted this assumption, it was impossible to use the TT model to accurately predict the germination rates and the time courses of germination across all the suboptimal temperatures (Figures 1A and B). We speculated that the general TT model was not applicable for the aspen seeds and required modification or be replaced with other models.

In aspen seeds, germination rates at 5°C were obviously higher than those predicted by the TT model (Figure 1A). This can be also observed in some species, like *Orobanche aegyptiaca* [19] and *Stellaria media* [33] seeds. Several studies have reported that the TT model had underestimated the germination rates and generated relatively large errors in the estimation of germination rates at some low temperatures [14], [22], [24]. Empirical models have been proposed to characterize the germination behavior at these temperatures [14], [22], [24], [25]. In some tropical and subtropical legumes, a quadratic equation can be used to closely match the change in germination rates with temperature [24]. In aspen seeds, this equation also fits well to the trend of a change in germination rates (data not shown). However, this model cannot provide any physiological interpretation. Some authors have suggested a piece-wise model using a series of step-wise linear equations to describe the change in germination rates with temperature [14], [22], [24], [25]. This model can increase the accuracy of germination predictions, but it is computationally complex and requires a large number of model parameters [24]. For the purpose of ecological modeling of seed germination, the empirical model may be satisfactory [34]. However, if we want to investigate further physiological and biochemical mechanisms controlling seed germination, a physiologically based model is necessary [13].

We observed that final germination percentages of aspen seeds increased with increasing temperature up to 15°C, and kept relatively constant from 15 to 30°C (Supplemental Figure S1A). This raises the question whether the dormancy was being imposed or the seeds were being damaged when germinated at the low temperatures and that influences the accuracy of the TT model. When the non-germinated seeds at different temperatures were stained with TTC to test viability, it was found that all of these seeds were dead (data not shown). This indicates that incubation of aspen seeds at the low temperatures (less than 15°C) does not induce dormancy, but has a detrimental effect on germination. Therefore, our hypothesis is that germination response of aspen seed to temperature is different between at the low temperatures (<15°C) and the high temperatures (≥15°C). Based upon this hypothesis, the MTT model was developed and was proven to predict relatively accurately germination rates and time courses of germination (Figures 1C and D). We believe that the sensitivity of germination to the low temperature is also a population based phenomena. Thus, we used a normally distributed *T_m_*(*g*) to account for the germination behavior at the low temperatures. The variation in *T_m_*(*g*) values indicates that the higher the percentage of seed to be germinated, the higher the temperature required, which accords with the change in final germination percentage with temperature (Supplemental Figure S1A). Increased thermal response of germination in the suboptimal range of temperature is generally attributed to the increased thermal activity of molecules involved in metabolic processes [11], [22], [35]. We speculate that the low temperature at which the aspen seed is germinated may affect the thermal activity of those molecules involved in germination processes.

To a certain extent, the MTT model is similar to the piece-wise model [22]. The latter model suggests replacing the suboptimal linear assumption of TT model with a series of separate equations for each a given temperature increment, for example, 5°C [14], [22], [24], [25]. In aspen seeds, it was evident that germination rates from 15 to 30°C followed a linear relationship with temperature (Figure 1B) and the TT model accurately predicted the percentages of germination at these temperatures (Figure 1D). Thus, it is no necessary to apply a lot of probit equations to model germination in this temperature range. The MTT model expands the temperature interval and reduces the equations and parameters used in the piece-wise model as well as the computational complexity. This indicates that the piece-wise model can be simplified to be more conveniently used in some species.

As other *Populus* species [5], [8], [26], [27], *P. tomentosa* seeds stored under ambient conditions can only survive for a relatively short time (Supplemental Figure S1B). The MLT model developed by Bradford et al [28] has been successfully applied to predict the germination percentage and rates of lettuce seeds after storage. In this study, we have proved that the MLT model can also describe the effect of storage time on the germination of aspen seed. The application of the MLT model is independent of germination temperature, in spite of the fact that it underestimates the final percentage of the seed germination (Supplemental Figure S1A).

Bradford et al [28] had proposed that the thermal time could be introduced into the MLT model to integrate a parameter of ‘aging thermal time’ to describe the combined effect of temperature and storage time on seed germination. This combination was evaluated by the ATT model in this study. As the TT model, a single set of model constants was not adequate to fit entire germination data across all the suboptimal temperatures and storage times (Figures 4A and C). This is mainly due to the different thermal response of germination between in low and high temperature ranges. By accounting separately for germination behavior at the low and high temperatures, the ATT model described well the effect of combined factor of temperature and storage time on seed germination (Figures 4B and D).

We have demonstrated that when temperature lowers to a given *T_i_*, the *p_max_*(*g*)s shift to lower values (Figure 3D). This change, acting as the *T_m_*(*g*), adjusts automatically the percentage of seed to be germinated under the cold temperature. It was mentioned above that the final germination percentage of control seeds decreased as temperature decreased in the low range (Supplemental Figure S1A). However, when we normalized the final germination percentage of control seeds at each temperature to 100% and fit the MLT model with the normalized data across all the storage times, it was found that the *p_max_*(*g*) values had small variations and kept relatively constant at all the germination temperatures (Supplemental Figure S2). These data imply that changes in *p_max_*(*g*) can be responsible for the sensitivity of germination to the low temperature. Physiologically, storage has a detrimental effect analogous to cold temperature on seed germination. We speculate that the sensitivities of germination to cold temperature and storage are possibly due to absence of a common defense mechanism against environmental stress.

As the germination rates, the change of 1*/θ_A_* with temperature was better fit with two different linear lines than only one linear line (Figures 3A and B). This explains further that germination at low and high temperatures has different physiological bases. Though 1/*θ_A_* can also be well related to temperature with a quadratic nonlinear model (Supplemental Figure S3), it appears that the value predicted from this nonlinear model has little difference from that predicted from the two-phased linear model, and the physiological meaning of the parameters in the nonlinear model is not such clear as those in the two-phased linear model. Thus, we believe that the two-phased linear model is preferable for aspen seeds.

### Conclusions

In *P. tomentosa seeds*, increase in germination rate with temperature in the suboptimal range was better fit to two different than only one linear equation, and consequently, seed germination was more accurately predicted with two different (MTT model) than with only one (TT model) probit equation. The MLT model described well the effect of storage time on seed germination across all the germination temperatures. When the ATT model combined the TT and MLT model was applied to fit germination data across all the germination temperatures and storage times, this model gave a relatively poor fitting to the germination data. As the TT model, adjusting the model to fit separately germination data at low and high temperatures in the suboptimal range increased largely the accuracy of the ATT model in germination prediction. The MTT and ATT models reflect that germination of aspen seeds is sensitive to both the cold temperature and increasing storage time. Thus, the aspen seeds, on the one hand, will suffer the damage of cold temperature on germination; on the other hand, will lose viability during storage under the natural condition after dispersion. Both effects decrease the probability of seed to be germinated. Above models indicate that physiological response of germination to temperature is distinct within suboptimal range.

This study presented with mathematic methods to quantify the effects of temperature, storage time and their combination on germination of aspen seeds. It is well known that the aging process depends on storage temperature and moisture. Further studies on the effects of these two factors on seed germination may be useful to predict more accurately germination of aspen seeds under the natural environment.

## Supporting Information

Figure S1 **Change in final germination percentage of control seeds with germination temperature (A) and in seed viability during storage (B).** The final germination was recorded after germination for 20 days at 5 and 10°C and for 7 days at 15–30°C. After that time, no germination of seeds was observed within 7 days. Change in seed viability with storage time was fit to a normal distribution equation (*y* = Ф(*μ*-*x*)/*σ*).
(TIF)

Figure S2 **Change of** ***p_max_*** **(** ***g*** **)s with temperature.** The germination data at each temperature across all the storage times were normalized on a basis of maximum germination percentage at that temperature. This normalized the final germination percentage of control seeds at all the temperatures to be 100%. The *p_max_*(*g*) was estimated after fitting the MLT model to the normalized data (solid circle) or the observed data (empty circle). *p_max_*(*g*)s were calculated for 10 (A), 30 (B), 50 (C) and 70% (D).
(TIF)

Figure S3 **Relationship between** ***1/θ_A_*** **and temperature.** *1/θ_A_* was related to temperature with a two-phased linear model (solid line, R^2^ = 0.996) or a quadratic nonlinear model (dashed line, R^2^ = 0.998).
(TIF)

Table S1 **Comparison between TT and MTT models in describing seed germination at various suboptimal temperatures after stored.**
(DOC)
