# Supplementary material for: Development of a Threshold Model to Predict Germination of Populus tomentosa Seeds after Harvest and Storage under Ambient Condition
Source: PLoS One. 2013 Apr 26;8(4):e62868. doi: 10.1371/journal.pone.0062868 (PMC3637295; doi:10.1371/journal.pone.0062868)
Supplement: Table S1 — Comparison between TT and MTT models in describing seed germination at various suboptimal temperatures after stored. (DOC) [file pone.0062868.s004.doc]

**Supplemental Table S1 Comparison between TT and MTT models in describing seed germination at various suboptimal temperatures after stored.**

|  | Model | Storage time (days) | | | | | | | | | | | |
| --- | --- | --- | --- | --- | --- | --- | --- | --- | --- | --- | --- | --- | --- |
|  |  | 0 | | 5 | | 10 | | 15 | | 20 | | 25 | |
|  |  | *R^2^* | *RMSE* | *R^2^* | *RMSE* | *R^2^* | *RMSE* | *R^2^* | *RMSE* | *R^2^* | *RMSE* | *R^2^* | *RMSE* |
| 5–30^o^C | TT | 0.86 | 0.136 | 0.82 | 0.140 | 0.78 | 0.130 | 0.82 | 0.116 | 0.80 | 0.092 | 0.77 | 0.089 |
|  | MTT | 0.95 | 0.081 | 0.89 | 0.107 | 0.89 | 0.095 | 0.89 | 0.090 | 0.77 | 0.099 | 0.76 | 0.092 |
| 5–10^o^C | TT | 0.77 | 0.129 | 0.83 | 0.120 | 0.37 | 0.183 | 0.61 | 0.139 | 0.56 | 0.111 | 0.72 | 0.091 |
|  | MTT | 0.99 | 0.029 | 0.82 | 0.125 | 0.89 | 0.078 | 0.88 | 0.077 | 0.82 | 0.071 | 0.94 | 0.041 |
| 15–30^o^C | TT | 0.88 | 0.139 | 0.81 | 0.149 | 0.88 | 0.101 | 0.86 | 0.106 | 0.86 | 0.083 | 0.79 | 0.089 |
|  | MTT | 0.94 | 0.098 | 0.92 | 0.096 | 0.89 | 0.101 | 0.90 | 0.095 | 0.76 | 0.109 | 0.70 | 0.107 |

TT and MTT models are used to fit the complete germination data at suboptimal range of temperatures for a given stored period (0, 5, 10, 15, 20 and 25 days), and the *R^2^* and *RMSE* are calculated for each model to compare the goodness of fit. Due to absence of enough data to fit the models, only results from 0–25 days are shown.
